# Neural Activity Changes Associated with Impulsive Responding in the Sustained Attention to Response Task

**DOI:** 10.1371/journal.pone.0067391

**Published:** 2013-06-25

**Authors:** Hiroyuki Sakai, Yuji Uchiyama, Duk Shin, Masamichi J. Hayashi, Norihiro Sadato

**Affiliations:** 1 Frontier Research Center, Toyota Central Research and Development Laboratories, Inc., Nagakute, Japan; 2 Precision and Intelligence Laboratory, Tokyo Institute of Technology, Yokohama, Japan; 3 Institute of Biomedicine, Physiology, University of Helsinki, Helsinki, Finland; 4 Brain Research Unit, O. V. Lounasmaa Laboratory, Aalto University School of Science, Espoo, Finland; 5 Institute of Cognitive Neuroscience, University College London, London, United Kingdom; 6 Division of Cerebral Integration, National Institute for Physiological Sciences, Okazaki, Japan; Baycrest Hospital, Canada

## Abstract

Humans can anticipate and prepare for uncertainties to achieve a goal. However, it is difficult to maintain this effort over a prolonged period of time. Inappropriate behavior is impulsively (or mindlessly) activated by an external trigger, which can result in serious consequences such as traffic crashes. Thus, we examined the neural mechanisms underlying such impulsive responding using functional magnetic resonance imaging (fMRI). Twenty-two participants performed a block-designed sustained attention to response task (SART), where each task block was composed of consecutive Go trials followed by a NoGo trial at the end. This task configuration enabled us to measure compromised preparation for NoGo trials during Go responses using reduced Go reaction times. Accordingly, parametric modulation analysis was conducted on fMRI data using block-based mean Go reaction times as an online marker of impulsive responding in the SART. We found that activity in the right dorsolateral prefrontal cortex (DLPFC) and the bilateral intraparietal sulcus (IPS) was positively modulated with mean Go reaction times. In addition, activity in the medial prefrontal cortex (MPFC) and the posterior cingulate cortex (PCC) was negatively modulated with mean Go reaction times, albeit statistically weakly. Taken together, spontaneously reduced activity in the right DLPFC and the IPS and spontaneously elevated activity in the MPFC and the PCC were associated with impulsive responding in the SART. These results suggest that such a spontaneous transition of brain activity pattern results in impulsive responding in monotonous situations, which in turn, might cause human errors in actual work environments.

## Introduction

Humans can plan and execute actions in preparation for uncertainties about upcoming external events to be handled, such as slowing down a vehicle at intersections with poor visibility to avoid collisions with crossing pedestrians or vehicles. It is difficult, however, to maintain this effort for every rarely occurring possibility over a prolonged period of time. Thus, people often impulsively (or mindlessly) respond in a habitual manner regardless of task or situational demands [1], [2]. Although impulsive responding might be somewhat beneficial for saving cognitive resources, it is recognized as a risk factor for serious accidents, including traffic crashes [3]–[5], medical accidents [6] and work-related injury [7]. To prevent such accidents from a neuroergonomic perspective, it is imperative to understand the underlying neural mechanisms of impulsive responding.

Impulsive responding can often result in behavioral errors. Therefore, maladaptive changes in neural activity preceding error responses in goal-directed tasks might be informative with respect to impulsive responding. Weissman et al. [8] reported that lapses in a local/global task are associated with reduced prestimulus activity in the anterior cingulate cortex (ACC) and the right prefrontal cortex (PFC). Furthermore, Eichele et al. [9] performed independent component analysis on functional magnetic resonance imaging (fMRI) data during a flanker task, and extracted a meaningful component exhibiting gradually reduced activity preceding errors in the presupplementary motor area (pre-SMA) and the right inferior frontal gyrus (IFG). Collectively, these results suggest that spontaneously reduced activity in such frontal control regions during task performance is a candidate neural mechanism for impulsive responding. However, since impulsive responding is not the only cause of errors in goal-directed tasks [10], it is insufficient (a logical fallacy) to identify the underlying neural mechanisms of impulsive responding by exploring only neural activity changes preceding errors. To overcome this problem, an online marker of impulsive responding is required.

In psychological studies, the sustained attention to response task (SART) has been widely used to assess the vulnerability of sustained attention [11], [12]. The SART is a Go/NoGo task where a NoGo stimulus is used at a lower frequency than Go stimuli, which requires participants to make Go responses under preparation for unpredictable NoGo responses. In fact, there is evidence that a patient population with difficulty in sustaining attention exhibited frequent NoGo errors when compared with normal controls [13], [14]. More importantly, it was demonstrated that Go responses followed by NoGo error responses tend to be faster than those followed by NoGo correct responses [11], [12], [15]. These data suggest that reduced Go reaction times may be a reasonable online marker of impulsive responding in the SART [11], [16]. Although a few fMRI studies have been reported using the SART [17], [18], there is no evidence of neural activity changes associated with the variability of Go reaction times.

Thus, the aim of the present study was to examine changes in neural activity associated with impulsive responding characterized by reduced Go reaction times in the SART using a block-designed fMRI paradigm. Each task block was composed of consecutive Go trials followed by a NoGo trial at the end. This experimental configuration enabled us to examine Go-related activity separately from NoGo-related activity because each task block consisted only of Go trials except for the last trial. We then conducted a parametric modulation analysis using block-based mean Go reaction times to identify neural activity changes associated with impulsive responding in the SART to provide new insight into the emergence mechanism of impulsive responding in monotonous work environments.

## Materials and Methods

### Participants

Twenty-two healthy subjects (12 males and 10 females, mean age of 26±7 years) participated in this study. All participants had normal or corrected-to-normal vision and were right-handed according to the Edinburgh Handedness Inventory [19]. This study was approved by the Ethical Committee of the National Institute for Physiological Sciences. Written informed consent was obtained from each participant after a full explanation of the study.

### Procedure

**Figure 1:**
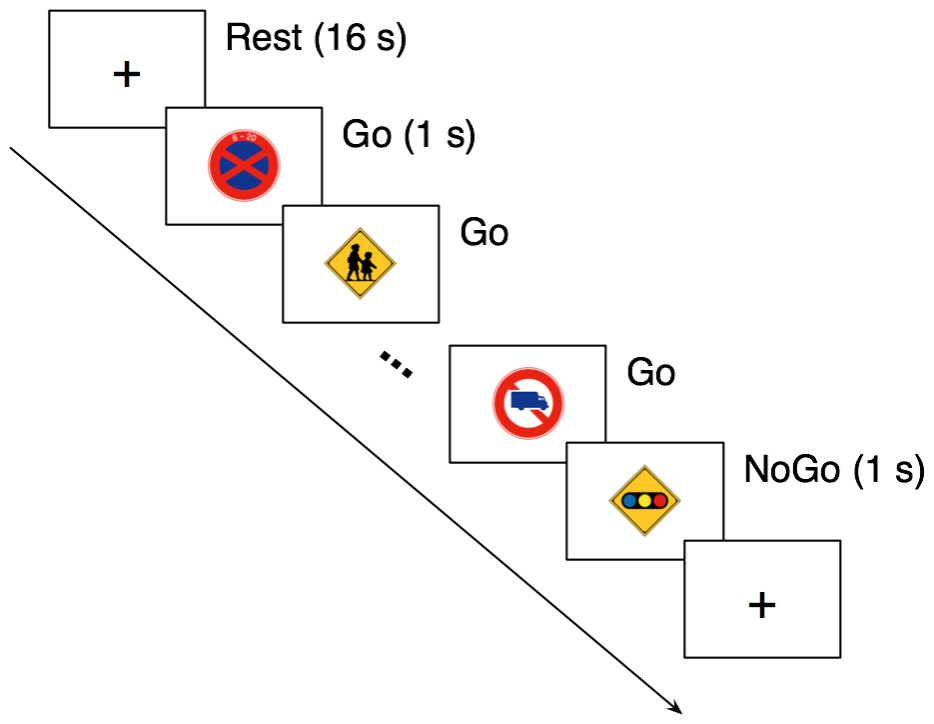
Timeline of the present sustained attention to response task. Following a fixation cross of 16 s (Rest), a sequence of Japanese traffic signs was presented in random order with a fixed duration of 1 s. The sequence length was varied from 8 to 52 s with a step of 4 s. The last stimulus of the sequence was always the sign warning a traffic light ahead. Participants were instructed to press a hand-held button for each sign (Go trial) except for the last one (NoGo trial).

A block-designed SART (Figure 1) was performed. Stimuli consisted of pictures of sixty Japanese traffic signs, and were displayed on a back-projection screen using an LCD projector DLA-M200L (Victor, Yokoyama, Japan). Following a fixation cross of 16 s as a rest block, a sequence of Japanese traffic signs was abruptly presented in random order with a fixed duration of 1 s, provided that the last stimulus of the sequence was always the sign warning a traffic light ahead. The sequence length was randomly varied from 8 to 52 s with a step of 4 s. Participants were instructed to press a button as soon as possible in response to each traffic sign with their right thumb (Go trial), and to withhold the response to the sign warning a traffic light ahead (NoGo trial). The task block was repeated 12 times in each run. Participants performed four runs (48 task blocks) in total. For each Go trial, a reaction time was measured as an interval between the stimulus presentation onset and the button-press response. For NoGo trials, error responses were defined as a button-press response within 1 s after the onset of a NoGo stimulus. By contrast, correct responses were defined as no response within the same time window. Stimulus presentation and response measurement were controlled using the software Presentation (Neurobehavioral Systems, San Francisco, CA, USA).

### fMRI Data Acquisition

The experiment was performed in a Siemens Allegra 3-T scanner (Siemens, Erlangen, Germany). For functional imaging, echo planar imaging (EPI) images were acquired using a T2*-weighted gradient echo sequence with a repetition time of 2 s, an echo time of 30 ms, a flip angle of 80°, a field of view of 192 × 192 mm and a matrix size of 64 × 64. Each EPI image comprised 34 contiguous 4 mm-thick slices with an in-plane resolution of 3 × 3 mm, and was positioned to cover the entire brain. During each of four runs, 289 images were collected (approximately 9.6 min), and the first five images were discarded to avoid the T1 saturation effect.

### fMRI Data Preprocessing

Image preprocessing (and the following statistical analysis) was performed using SPM8 (Wellcome Department of Cognitive Neurology, London, UK). EPI images in each run were realigned to the first image to correct head motion, and then spatially normalized to a standard EPI template from the Montreal Neurological Institute (MNI). After normalization, the images were resampled to a voxel size of 2 × 2 × 2 mm, and finally smoothed by convolution with a 6 × 6 × 8 mm full-width-at-half-maximum Gaussian kernel. Low frequency drifts in the time series of EPI images were removed by applying a high-pass filter with a cut-off of 256 s.

### Data Analysis

In the preset study, we assumed that reduced Go reaction times were an online marker of impulsive responding in the SART. In fact, several lines of evidence have demonstrated that Go reaction times preceding NoGo commission errors tend to be shorter than those preceding NoGo correct responses [11], [12], [15]. To validate our assumption, however, it was more essential to show that relatively faster Go responses tended to be followed by NoGo errors. Therefore, we examined whether block-based mean Go reaction times were capable of predicting NoGo errors using the receiver operating characteristic (ROC) analysis. For each task block, the NoGo response was predicted as an error when the mean Go reaction time was shorter than a threshold value. A ROC curve was then constructed by varying the threshold value. Finally, predictive accuracy was quantified as the area under the ROC curve for each participant, and statistically examined with a one-sample t-test to show better performance than a completely random guess (accuracy of 0.5).

For individual participant analysis of fMRI data, a general linear model was applied voxel-wise to the time series of EPI images. Task specific effects were estimated with two regressors: one was generated by convolving a canonical hemodynamic response function into a commonly-used box-car function representing the task design; the other was generated into the same box-car function, provided that its height in each task block was parametrically modulated with the mean Go reaction time. By contrasting the task and rest epochs, the first regressor would identify Go-related activity, which was of no interest in our study (results are shown in Supporting Information, Figure S1 in File S1), while the second would identify brain regions associated with Go reaction time variability, which was, in turn, associated with impulsive responding in the SART. Subsequently, for group analysis, a random effects model was applied voxel-wise to the individual contrast images for each task specific effect. The statistical criterion was set to uncorrected *P*<0.001 at the voxel level with corrected *P*<0.05 for multiple comparisons using family-wise error at the cluster level. After the group analysis, we searched for the local maximum in each significant cluster.

We also examined neural activity changes preceding NoGo commission errors in the SART. Go-related activity in the blocks where NoGo error responses were observed was contrasted with that in the blocks where NoGo correct responses were observed using identical statistical criteria to the parametric modulation analysis (uncorrected *P*<0.001 at the voxel level with corrected *P*<0.05 for multiple comparisons at the cluster level). In this analysis, however, four participants who made all the correct or error responses in every NoGo trial in either run were excluded. The results of this analysis supported that the use of reduced Go reaction time as a behavioral marker of impulsive responding in the present study provides a unique measure when compared with previous studies examining maladaptive neural activity changes preceding errors in goal-directed tasks.

## Results

### Behavioral Data

**Figure 2:**
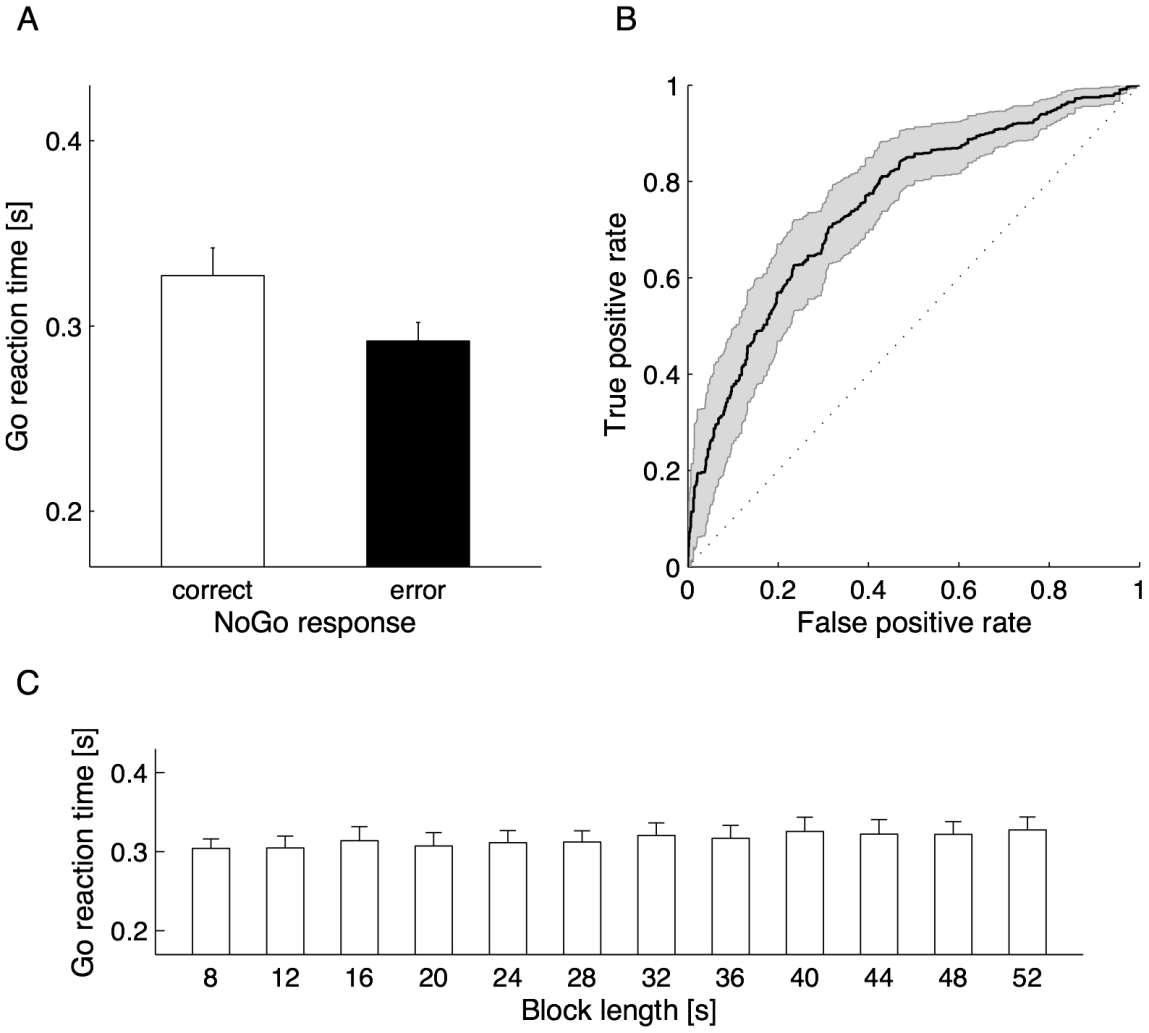
Behavioral data. Go reaction times preceding NoGo correct (open bar) and error (filled bar) responses were compared (A). In addition, the receiver operating characteristic analysis was made to examine whether block-based mean Go reaction times were capable of predicting NoGo errors (B). Go reaction times were further compared to test the impact of block length as a possible confounding factor (C). Error bars in the panel A and C and shaded area in the panel C represent the standard error across participants.

Commission errors were observed in 39±25% (range, 4–83%) of NoGo trials across participants. Consistently with previous SART studies [11], [12], [15], Go reaction times preceding NoGo error responses were significantly shorter than those preceding NoGo correct responses (*P*<0.001, paired t-test; Figure 2A). More importantly, ROC analysis revealed that the predictive accuracy for NoGo errors by block-based mean Go reaction times was significantly better than a completely random guess (0.76±0.12, *P*<0.001, one-sample t-test; Figure 2B). These data support our assumption that block-based mean Go reaction times are a suitable online measure of impulsive responding in the SART.

In addition, we performed a repeated-measures analysis of variance on mean Go reaction times as a function of block length to examine a possible confounding effect on the following fMRI data analysis. As a result, there was no significant main effect of block length on mean Go reaction times (*P*>0.1; Figure 2C), suggesting that block length was not a confounding factor in our fMRI data analysis.

### fMRI Data

**Figure 3:**
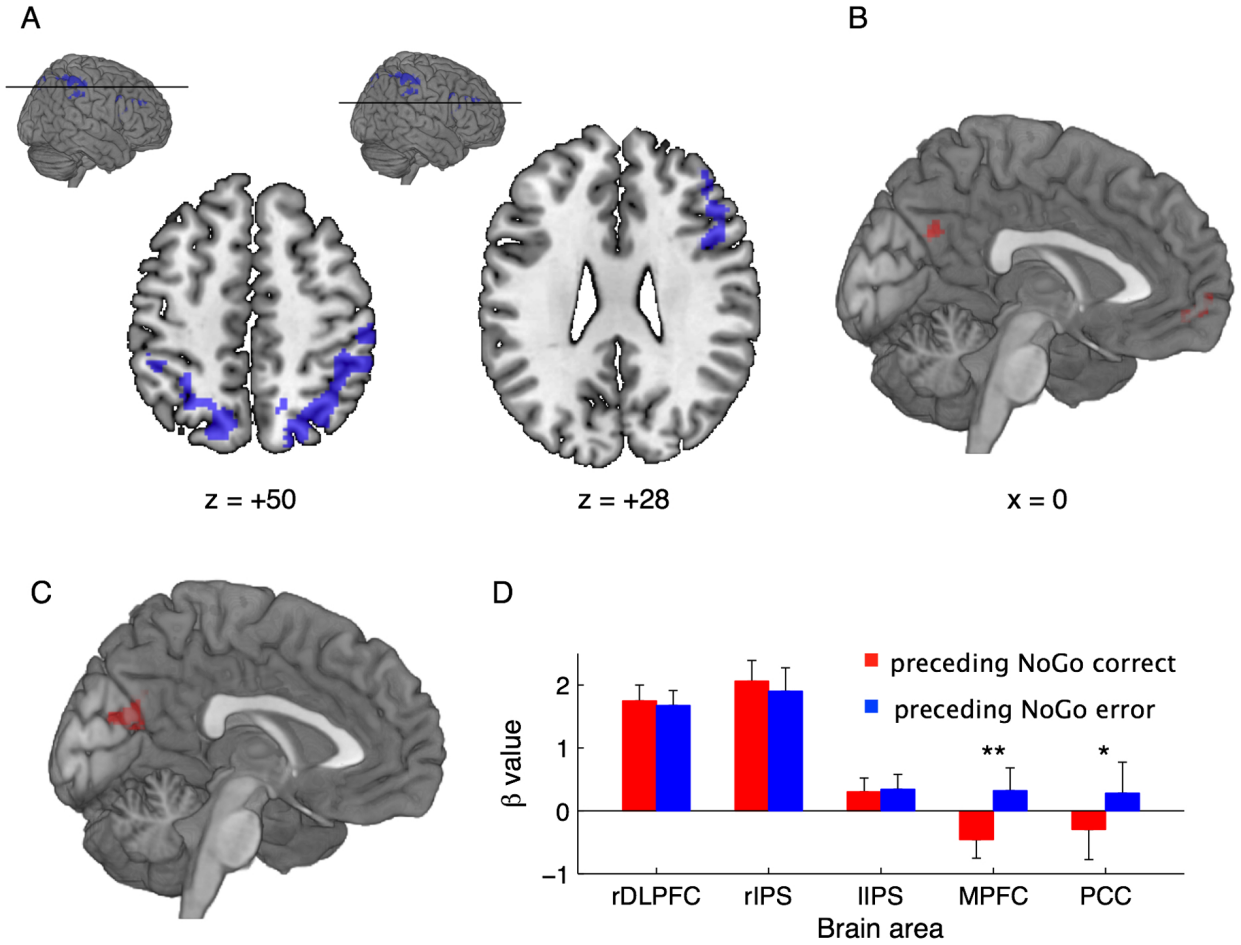
fMRI data. Parametric modulation analysis identified brain regions whose activity in each task block was positively (A) and negatively (B) correlated with the block-based mean Go reaction time. In addition, by exploring brain regions showing greater activity preceding NoGo errors than preceding NoGo corrects, a significant cluster was found in the medial posterior part of the brain (C). A statistical criterion was commonly set to uncorrected *P*<0.001 at a voxel level with a family-wise error-corrected *P*<0.05 for multiple comparisons at cluster level, provided that the multiple comparison correction was not applied for the result of negative correlation. Moreover, activity differences between preceding NoGo correct and error responses were examined at the peak loci extracted in the parametric modulation analysis (D).

Brain regions whose activity in each task block was positively correlated with the mean Go reaction time were found in frontal and parietal areas (Figure 3A; also see Supporting Information, Table S1 in File S1). The cluster within the frontal areas extended to the right inferior (BA 9/44/45) and the middle frontal (BA 9/46) gyri, while the other two clusters within the parietal areas extended to along the bilateral intraparietal sulcus (IPS), including the precuneus (BA 7), the superior (BA 7) and inferior (BA 40) parietal lobes. This positive correlation between neural activity and the mean Go reaction time during task performance suggests that spontaneously reduced activity within the right dorsolateral PFC (DLPFC) and the bilateral IPS is associated with impulsive responding in the SART. Meanwhile, activity in each task block that was negatively correlated with the mean Go reaction time was not significant under our statistical criteria corrected for multiple comparisons. However, the largest two clusters were found along the medial wall of the hemisphere (Figure 3B; also see Table S1 in File S1); i.e., the medial PFC (MPFC, BA 10) and the posterior cingulate cortex (PCC, BA7/31). These neural activities significantly associated with Go reaction time variability were further validated using a different analytical method (see Supporting Information, Text S1 and Figure S2 in File S1, for details).

We also investigated neural activity changes preceding NoGo errors. Under our statistical criteria, no area showed significantly reduced activity preceding NoGo error responses when compared with preceding NoGo correct responses, while increased activity was detected in the medial posterior part of the brain including the PCC and the cuneus (Figure 3C; also see Table S1 in File S1). Additionally, activity differences between preceding NoGo correct and error responses were tested at the peak loci extracted in the aforementioned parametric modulation analysis. Consequently, the loci that were positively correlated with Go reaction times showed no significant activity changes (*P*>0.1, paired t test; Figure 3D), while the loci that were negatively correlated showed greater activity preceding NoGo error responses when compared with preceding NoGo correct responses (*P*<0.05, paired t test; Figure 3D).

## Discussion

Behavioral data analysis revealed that in the SART, Go reaction times were capable of predicting NoGo commission errors, suggesting that responses occasionally become too reflexive to be withheld in NoGo trials. Hence, as we assumed, reduced Go reaction time can be used as an online marker of impulsive responding. By parametric modulation analysis of fMRI data using block-based mean Go reaction times, we found that spontaneously reduced activity within the right DLPFC and the bilateral IPS and (albeit statistically weak) spontaneously elevated activity within the MPFC and the PCC were associated with impulsive responding in the SART.

Shorter reaction times in cognitive tasks are widely used as a behavioral measure of facilitative effects of attention on task-relevant sensory information processing. For instance, shorter reaction times in the Posner paradigm [20] have been considered as a consequence of facilitated visual processing at stimulus locations to which visual attention is oriented. Under this operational definition of attention, our behavioral data can be interpreted as that excessive attention to visual stimuli enhances response speed over accuracy in Go trials, and therefore results in the failure of (reactive) inhibitory control to withhold an impulsive response to a NoGo stimulus. Accordingly, spontaneously reduced activity within the right DLPFC and the bilateral IPS during SART performance can be associated with the engagement of excessive visual attention. However, there is abundant evidence demonstrating that those frontoparietal regions construct a neural network that has a pivotal role in endogenous attention control ([21], [22] for the right DLPFC; [23]–[28] for IPS; see [29], [30] for reviews). In other words, this frontoparietal network becomes active when attention is voluntarily engaged in the task in hand. Thus, we speculate that spontaneously reduced activity in the frontoparietal regions during SART performance is associated with the transient deterioration of attention rather than the facilitative effects of attention, which totally contradicts the interpretation of our fMRI data derived from the commonly used operational definition of attention. Conversely, supposing that impulsive responding characterized by reduced Go reaction times represents the transient deterioration of attention during SART performance, then our fMRI data are consistent with previous neuroimaging studies of endogenous attention control. However, our behavioral data are inconsistent with previous behavioral studies demonstrating that decreasing attention, as typified by drowsiness, causes response slowing in general [31], [32].

Proactive inhibitory control, a relatively new concept in cognitive neuroscience studies focusing on the intentional withholding of a motor response (see [33] for review), might be able to provide an alternative explanation for impulsive responding in the SART. Although inhibitory control (or response inhibition) is one of the most examined type of executive function, the majority of studies focus on the reactive aspects; i.e., inhibitory control of responses that have already been initiated in response to an external triggering event. Compared with this, proactive inhibitory control functions as a brake whenever uncertainties are expected in upcoming events; i.e., enabling slow but accurate responses. As proactive inhibitory control is compromised, responses can be triggered automatically by external events regardless of their contents. This is a possible cause of our behavioral data that faster Go responses tend to be followed by NoGo commission errors in the SART. Jaffard et al. [34] suggested that proactive inhibitory control is maintained at least for several seconds. Our behavioral data additionally indicate that proactive inhibitory control is vulnerable in monotonous situations that last for a few tens of seconds.

Under this cognitive model, our fMRI data can be interpreted as that the right DLPFC and the IPS are involved in proactive inhibitory control. Additionally, our data suggest that the neural substrate of proactive inhibitory control largely overlaps with that of endogenous attention control. Although there is little evidence of the underlying mechanisms of proactive inhibitory control, Jaffard et al. [34] reported elevated activity in some cortical and subcortical regions during a detection task in which proactive inhibitory control was required, when compared with during a simple (pure) detection task that allows reflexive responses without proactive inhibitory control. In that study, the cortical regions included the superior parietal lobule and the precuneus, which are consistent with our interpretation that activity in the parietal cortex along the IPS was reduced as proactive inhibitory control was compromised. More recently, however, Zandbelt and colleagues [35], [36] concluded that the striatum is a critical node in the neural network associated with anticipation of response stopping. In the present study, no subcortical regions including the striatum showed significant associations with the variability of Go reaction times. By contrast, there is limited evidence for a role of the right DLPFC in the context of proactive inhibitory control. Although the vulnerability of proactive inhibitory control is a plausible mechanism for the emergence of impulsive responding, further empirical evidence is required.

In the present study, we found that reduced Go reaction times were predictive of NoGo commission errors. Nevertheless, we did not find a significant reduction in activity preceding errors in brain regions whose activity positively correlated with Go reaction times. This apparent dissociation suggests that reduced Go reaction times, used as an online marker of impulsive responding in our study, reflected a separate aspect of erroneous behavior in the SART from NoGo commission errors. NoGo errors can occur because of not only anticipatory processes compromised during Go responses but also the failure of reactive processes in response to NoGo stimuli. Therefore, multiple factors might influence neural activity changes preceding errors in the present study, which makes it difficult to identify specific brain regions significantly associated with NoGo errors. By contrast, previous studies examining maladaptive activity changes in goal-directed tasks [8], [9] reported that activity was reduced preceding errors in some frontal regions such as the ACC, the pre-SMA and the right IFG. Compared with the present SART, cognitive tasks employed in those studies provided response conflicts more frequently, and as such, reactive inhibitory control might be a dominant factor to determine whether or not errors are made. In fact, those frontal regions are known to play a critical role in reactive inhibitory control ([37]–[42] for ACC; [43]–[46] for pre-SMA; [43]–[48] for right IFG). Accordingly, reduced activity preceding errors within the frontal control regions previously observed might be a neural signature (or precursor) of the impairment of reactive, but not proactive, inhibitory control.

We also found a negative association between Go reaction time variability and neural activity in the medial regions of the brain (the MPFC and the PCC), although under somewhat liberal statistical criteria (no multiple comparison correction). These medial regions are known as a core of the default mode network (DMN) [49]. Thus, our results suggest that spontaneously elevated DMN activity during task performance is also associated with impulsive responding in the SART. Interestingly, unlike brain regions showing a positive association, the MPFC and the PCC were both more active preceding NoGo error responses than preceding NoGo correct responses. This is consistent with increasing evidence of interference of task-specific processing with spontaneously elevated DMN activity during task performance (see [50] for review). For instance, Cristoff et al. [16] reported that DMN activity increased preceding NoGo commission errors in the SART. Li et al. [51] also demonstrated that greater activity within the DMN could reliably predict commission errors in a stop-signal task, which was replicated by Eichele et al. [9] using a flanker task. In addition, there is evidence demonstrating that increased DMN activity is involved in the decoupling of attention from perceptual input or in mind wandering [16], [52]. Collectively, spontaneously elevated activity within the DMN might deteriorate SART performance in a general rather than specific manner.

In summary, our data suggest a possible neural mechanism underlying impulsive responding, as follows: when one deals with a goal-directed task or work, specific brain regions are engaged to accomplish the goal. In a situation where there is uncertainty in the upcoming event to be handled, the right DLPFC and the bilateral IPS play a critical role in maintaining preparation for the uncertainty. However, particularly in monotonous environments, the maintenance implemented in such frontoparietal regions is vulnerable, and as such, the brain tends to return toward the default mode. This spontaneous transition of brain activity pattern results in impulsive responding, which in turn might cause human errors in actual work environments.

## Supporting Information

File S1 (PDF)
