# Supplementary material for: Neural Activity Changes Associated with Impulsive Responding in the Sustained Attention to Response Task
Source: PLoS One. 2013 Jun 25;8(6):e67391. doi: 10.1371/journal.pone.0067391 (PMC3692459; doi:10.1371/journal.pone.0067391)
Supplement: File S1 — (PDF) [file pone.0067391.s001.pdf]

Figure S1.

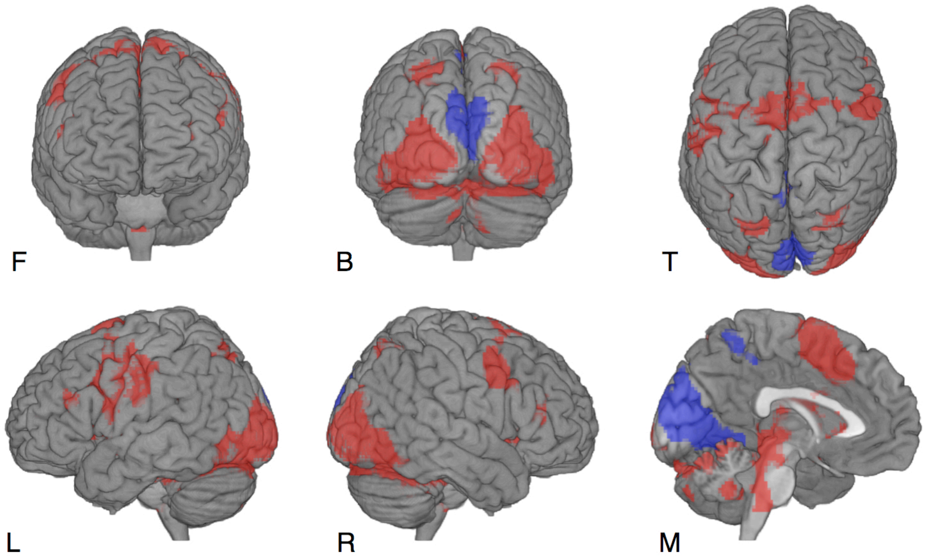

Figure S1. Go-related activation and deactivation in the present sustained attention to response task. A statistical criterion was set to uncorrected $P < 0.001$ at the voxel level, with family-wise error-corrected $P < 0.05$ for multiple comparisons at the cluster level. Go-related activation (red) was extensively distributed in cortical and subcortical regions, including occipital and temporal visual cortices (BA 17/18/19/37), superior and inferior parietal lobules (BA 7/40), left primary motor cortex (BA 4), premotor cortex (BA 6), supplementary motor area (BA 6), anterior cingulate cortex (BA 23/32), lateral frontal areas (BA 9/44/46), cerebellum, thalamus and brainstem. By contrast, Go-related deactivation (blue) was found in the medial posterior part of the brain (BA 7/18). F, B, L, R, T and M denote the front, back, left, right, top and medial view of the brain, respectively.

Table S1.

Positive correlation (Figure 3A)

Hemisphere	*Brain region*	*BA*	*Peak locus*	*Z-score*	*k*
RH	Prefrontal cortex	9/44/45/46	(44, 10, 28)	4.04	507
RH	Intraparietal sulcus	7/39/40	(30, -64, 48)	4.19	1907
LH	Intraparietal sulcus	7/40	(-12, -60, 50)	4.25	1171

Negative correlation (Figure 3B)

Hemisphere	*Brain region*	*BA*	*Peak locus*	*Z-score*	*k*
LH	Medial prefrontal cortex	10	(-6, 62, -2)	4.39	37†
LH	Posterior cingulate cortex	31	(0, 62, 30)	3.54	45†

NoGo error > NoGo correct (Figure 3C)

Hemisphere	*Brain region*	*BA*	*Peak locus*	*Z-score*	*k*
LH	Posterior cingulate cortex	31	(-12, -72, 18)	3.98	403
RH	Insula	13	(46, -10, 6)	4.21	227

Note. Coordinates of each peak locus refer to the local maxima within a significant cluster in the Montreal Neurological Institute coordinate space. BA denotes the Brodmann area. *k* represents cluster size. LH and RH refer to the left and right hemispheres, respectively. A statistical criterion was commonly set to uncorrected $P < 0.001$ at a the voxel level, with a family-wise error-corrected $P < 0.05$ for multiple comparisons at the cluster level, provided that the multiple comparison correction was not applied for the result of negative correlation, as indicated with daggers (†).

Text S1.

We performed parametric modulation analysis to identify brain regions whose activity in each task block was correlated with the mean Go reaction time. As a result, the right dorsolateral prefrontal cortex (DLPFC) and the bilateral intraparietal sulcus (IPS) were extracted as regions of positive correlations. In addition, the medial prefrontal cortex (MFPC) and the posterior cingulate cortex (PCC) were identified as regions of negative correlations. Here we verified these results by directly examining the correlation between activity in each task block and the mean Go reaction time at each peak locus extracted in the parametric modulation analysis (Table S1).

For this purpose, Go-related activity was modeled by convolving a box-car function with a canonical hemodynamic response function separately for each task block. Next, a correlation coefficient between beta values and the mean Go reaction times was computed at each peak locus. Finally, the mean correlation coefficient across participants was statistically tested using a one-sample t-test for each peak locus.

Consequently, the right DLPFC and the bilateral IPS showed positive correlations between activity in each task block and the mean Go reaction time, while the MPFC and the PCC showed negative correlations. Although these correlations were not so strong and varied across individuals and loci, we reconfirmed that the correlation coefficient was significantly deviated from zero in each peak locus (see Figure S2, below). This result corroborates the results of the parametric modulation analysis (Figure 3A and 3B).

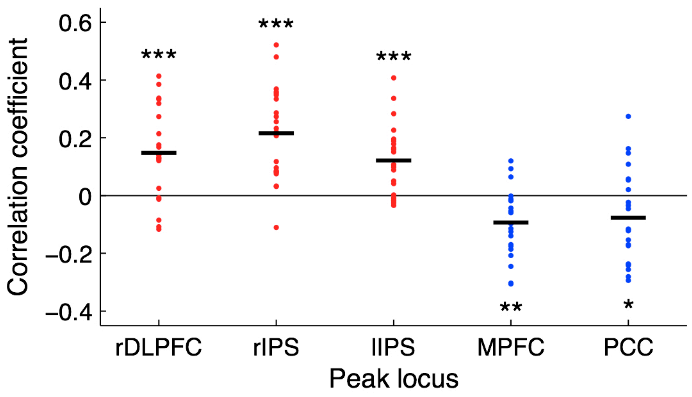

Figure S2. Correlation coefficient between Go-related activity and the mean Go reaction time at each peak locus extracted in the parametric modulation analysis. Each dot represents one participant. Short horizontal bars show the mean correlation coefficient across participants. Asterisks denote that the mean correlation coefficient was significantly deviated from zero (***: $P < 0.001$; **: $P < 0.01$; *: $P < 0.05$).
